# Male Reproductive Health: A village based study of camp attenders in rural India

**DOI:** 10.1186/1742-4755-1-7

**Published:** 2004-11-22

**Authors:** Kate M Dunn, Susmita Das, Rumeli Das

**Affiliations:** 1 Primary Care Sciences Research Centre, Keele University, Staffordshire, ST5 5BG, UK; 2 Family Health International (FHI), India Country Office, Opposite Convention Hall, Ashok Hotel, Chanakyapuri, New Delhi 110021, India; 3 Child in Need Institute (CINI), PO Pailan via Joka, Kolkata 700104. West Bengal, India

## Abstract

**Background:**

A paucity of information about male reproductive health and a perceived interest in involvement among local men provided the impetus for carrying out a village based male reproductive health camp. The aim was to investigate men's willingness to participate in such camps, and to describe reproductive health problems in men.

**Methods:**

Structured interviews were carried out with 120 men attending a reproductive health check-up in a village in rural West Bengal, India. General information, details of family planning methods used and data on reproductive health complaints were collected. Clinical examinations were also carried out. Socio-demographic characteristics were compared for men with and without reproductive health and urinary complaints.

**Results:**

Three quarters of the married men were using contraception, but the majority stated that their wives were responsible for it. The most common reproductive health complaint was urinary problems; 28% had burning on urination, and 22% reported frequent and/or difficult urination. There were few social or demographic differences between men with and without problems. Seventeen percent of the men had clinically diagnosed reproductive health problems, the most common being urethral discharge. None of the men with diagnosed problems were using condoms.

**Conclusions:**

This study highlights the interest of men in their reproductive health, but also highlights the high proportion of men with problems. In addition, a number of men with clinically diagnosed problems had not reported them in the interviews, illustrating either the reticence to report or the lack of knowledge about symptoms of reproductive health problems. Recommendations for future programmes and research in this field are given.

## Background

Reproductive health is a major world priority, with particular problems in developing countries. However, as Ndong [1] states, "reproductive health generally has been synonymous with women's health", and reproductive health of men has received little attention [2]. Researchers and health planners have pointed out that better outcomes for reproductive health programmes would be expected if men were involved [2-6], and there are a number of mechanisms by which this might occur. Hawkes [7] indicated that the treatment of male reproductive health problems might actually encourage more women to seek treatment, and therefore improve the overall level of reproductive health. Other studies have highlighted that women often need the support of their husbands, including financial support, to attend reproductive health services [4,8,9] and that the health status of couples, in particular the reproductive health status, is strongly linked to the knowledge, attitudes and behaviour of men [6,10].

The documentation of the prevalence of male reproductive health problems has been identified as a research priority by the World Health Organization (WHO) [5]. Until now limited information on reproductive health problems and health care needs in men from developing countries, particularly from rural areas of India, is available [11,12]. Verma [12] studied male sexual problems in a slum population in Mumbai (India) and found that around half of the men studied could identify symptoms such as itching, burning on urination and white discharge, although the prevalence of these symptoms among these men was not reported. One study from Bangladesh reported that over 10% of men had symptoms that were possibly indicative of a sexually transmitted infection, with the main complaints being urethral discharge and pain passing urine [2].

Information about male reproductive health problems may help in the planning of future services, the needs for screening, prevention and treatment. In order to collect this information, a village based reproductive health camp for men was carried out. Health camps are used to provide services in places where the perceived need and demand is high but access to treatment is poor due to lack of information about available services, distance from health facilities, or low economic status of the population. It is one of the preferred methods of service provision in rural villages. The usual model is a temporary clinic set up by a team of health personnel at a community-owned location (e.g. school or community hall) to provide services and medicines usually free of cost based on the health needs of the community. The aim of this study was to investigate men's willingness to participate in such camps, and to describe reproductive health problems in this group of men. This paper reports on the findings from this health camp.

## Methods

### Study participants

Men aged 15–60 years, both married and unmarried and living in one rural village were invited to attend. The village is relatively small (approximately 4000 residents), mostly consisting of low-income, Muslim population, and had been highlighted by local health workers as having particularly poor knowledge about reproductive health issues.

### Camp procedure

The camp was held in a village school on one day in February 2000. The community was consulted about the camp through a variety of routes, including men's groups, Panchayats (local self government), schoolteachers and specially convened public meetings; all of these groups agreed to the holding of the camp in the village. The camp was publicised through peer educators, health workers and group meetings, and was described as a reproductive health check-up, with free treatment if necessary. A male team was used as feedback from health workers and peer educators in the village indicated that men would be more willing to attend if male doctors carried out the examinations.

Health workers carried out a confidential interview on arrival to obtain demographic information (age, marital status, number of children, employment and income), information about personal hygiene, details about family planning issues and about general health complaints (including fever, dizziness, backache, headache, skin problems) by using a structured interview schedule. Male doctors then asked about reproductive health complaints (including urinary problems, genital itching, penile sores/swelling, urethral discharge) and current sexual activity (sex outside marriage or with a prostitute), and carried out clinical examinations (recording of urethral discharge, genital ulcer, inguinal lymph nodes, penile lesions, scrotal swelling, genital warts or scabies). Blood samples were taken for Venereal Disease Research Laboratory (VDRL) test from all attendees.

Those men detected as having sexually transmitted infections and urinary problems were given the appropriate medication, provided with condoms and referred to hospital when necessary. Advice and instructions for condom use were given and patients were encouraged to bring their partners for treatment.

### Setting

The Child in Need Institute (CINI) is a non-governmental organisation (NGO) working in West Bengal, and is one of the four national NGOs in India. The organisation has, for the last 30 years, focused on the health and nutrition of women and children, with the additional involvement of adolescents and men. CINI started working on reproductive health with men in rural areas in 1997. To date, this involvement has entailed orientation and education with men's groups, intensive training of voluntary peer educators and facilitators and individual interaction with health workers. There are at present no specific clinical services for men provided by CINI: men who are identified as having a problem are given individual information and counselling and are referred to other clinics for treatment.

### Analysis

The information collected during the interviews, clinical examination and pathology reports was entered into the Epi-Info data entry and analysis package [13]. Statistical analyses were carried out using the SPSS statistical package [14]. The relationship between reproductive health complaints, clinical findings and other health and socio-demographic factors was investigated. P-values for the differences between means were calculated using the independent samples t-test. P-values for the differences between proportions were calculated using the chi-squared test or Fisher's exact test.

## Results

One hundred and twenty men attended the camp; all men responded freely to the interview, and all but two agreed to a clinical examination. This sample represents about 10% of the local male population aged 15–60 years.

### Demographic information

The mean age in this group of men was 29.6 years (range 16–60). Fifty-nine percent of the men were married (n = 71); the mean age at marriage was 22.6 years (range 14–34). Fifty-two percent of the attendees had children (comprising 90% of the married men); among the men with children, the mean number was 2.8 (range 1–7). The literacy rate was 71% among the attendees, although more than half of these had only studied up to class V or less. Eighty four percent of the attendees (n = 85, data missing for 19 attendees) had a monthly income of less than Rs. 2000 (~$45); 40% (n = 48) were daily wage earners, and a further 30% (n = 36) were in service (usually unskilled office workers), 15% (n = 18) were either unemployed or students. One third of the men worked away from home for at least one night each month, half of these for 1–5 days, and half for more than 5 days per month. Only 11 attendees indicated that they consumed alcohol.

Twenty percent (n = 24) reported good personal hygiene (washing genital areas and changing underwear daily), however 28% (n = 33) had very poor hygiene practices (washing genital area and/or changing underwear less than twice a week).

### General health

Four fifths of men (82%) reported at least one problem with their general health, the most commonly reported problems were coughs and colds (45%, n = 54), and weakness (28%, n = 34), other problems included indigestion, backache and headache. Two men were identified as having hypertension.

### Family planning

Forty-three percent of all men (n = 51) reported that they or their wives were using some kind of family planning, this represented 73% of the married men; only one unmarried man was using contraception (condoms). Over half of the men stated that their wives are responsible for family planning, they are using the oral contraceptive pill, have an intrauterine device or had sterilisation. Eight men were using condoms, and one man had had a vasectomy. The main reason given for not using family planning was being unmarried. Two men reported a lack of knowledge of family planning, two men had religious objections and five men said that they wanted children.

### Sexual behaviour

Six married men admitted to having extramarital sex, three of them with prostitutes. Seven unmarried men (14%) reported having sexual relations; three of them with prostitutes. None of the men visiting prostitutes reported using condoms, however one married man having extra marital sex reported using condoms. It did not appear that the men visiting prostitutes or having extra-marital sex were any different from the other men in terms of monthly income, education or number of nights working away from home.

### Reproductive health complaints

Half of the attendees reported at least one reproductive health complaint (n = 60) (table 1). Only three men with a reproductive health complaint reported using condoms (table 2). There were no statistically significant differences in age, monthly income, days of absence from home or marital status between men with and without reporting of sexual health problems. However, men who have complaints have on average fewer years of education (3.3) compared to men who do not have complaints (4.9). Similar proportions of men were married in those with and without reproductive health complaints (table 2). The most common complaints were urinary problems, which were reported by 43% of attendees (n = 51); 28% (n = 33), of men complained of burning on urination and 23 % (n = 27) reported frequent and/or difficult urination.

**Table 1:** Self reported complaints of reproductive health problems

Complaint	No of attendees	% (n = 120)
Burning on urination	33	28%
Frequent urination	20	17%
Difficulty urinating	7	6%
Strong urine	4	3%
*Any urinary complaint*	*51*	*43%*
Premature ejaculation	6	5%
Urethral discharge	6	5%
Genital itching	5	4%
Impotence	2	2%
Primary infertility	2	2%
*Any reproductive health complaint*	*60*	*50%*

**Table 2:** Socio-demographic characteristics in men with and without reproductive health & urinary complaints

Demographic variable	Any reproductive health complaint			Burning on urination			Frequent and/or difficult urination		
	Yes n = 60	No n = 60	p-value	Yes n = 87	No n = 33	p-value	Yes n = 94	No n = 26	p-value
Age (mean years)	29.8	29.1	0.761^†^	28.3	29.9	0.498^†^	31.7	28.8	0.249^†^
Monthly income (mean rupees)	1447	1411	0.905^†^	1283	1493	0.528^†^	1146	1515	0.307^†^
Education (mean years)	3.3	4.9	0.009^†^	3.7	4.2	0.476^†^	3.2	4.3	0.126^†^
Time away from home (mean days per month)	2.8	1.9	0.330^†^	2.5	2.3	0.825^†^	3.6	2.0	0.141^†^
Marital status: no., (%) married	35 (58%)	34 (57%)	0.853^‡^	17 (52%)	52 (60%)	0.414^‡^	16 (62%)	53 (56%)	0.638^‡^
Condom use: no. (%) using condoms	3 (5%)	5 (8%)	0.717*	3 (5%)	5 (8%)	0.683*	1 (2%)	7 (12%)	1.000*
Sex with prostitute: no. (%) of men	2 (3%)	4 (7%)	0.679*	1 (2%)	5 (8%)	1.000*	0	6 (10%)	0.338*
Good personal hygiene: no. (%) of men**	13 (26%)	11 (19%)	0.492*	10 (32%)	14 (18%)	0.129*	4 (20%)	20 (23%)	1.000*

^† ^p-value for the difference between means using independent samples t-test; ^‡ ^p-value using chi-squared test

* p-value using Fisher's exact test; ** Reporting washing genital areas and changing underwear daily

### Clinical reproductive health findings

On clinical examination, 20 men (17%) had findings of reproductive health problems, (table 3), fourteen of these men had reported reproductive health complaints in the interview. None of the men with clinical findings reported using condoms, and nearly half (n = 9) had poor self-reported hygiene. Two of the men with positive findings reported visiting prostitutes, and five reported having extramarital sex. The most common clinical finding was urethral discharge, which was found in 13 men, this included all 6 men who had reported urethral discharge in the interview (table 4); two of these men reported having sex with a prostitute. Painful genital lesions were found in two men, this could be an indicator of the herpes virus; these men were both married and one was visiting a prostitute, neither used condoms and neither reported penile sores in the interview, although both reported genital itching (table 4). Only one man had a positive VDRL result, he had reported genital itching, the clinical examination found a genital ulcer and swollen and painful lymph nodes. He was married, and reported having extra marital sex with a prostitute, and said he did not use condoms.

**Table 3:** Clinical findings of reproductive health problems

Clinical sign	No of attendees	% (n = 118)
Urethral discharge	13	11%
Genital ulcer	2	2%
Swollen lymph nodes	5	4%
Painful genital lesions	2	2%
Scrotal swelling	0	-
Genital warts	0	-
Scabies	2	2%
Fungal infection	2	2%
*Any clinical sign*	*20*	*17%*

**Table 4:** Comparison of self reported complaints and clinical findings of reproductive health problems (no. of attendees)

	Clinical finding						
Self-reported complaint	Urethral discharge (n = 13)	Genital ulcer (n = 2)	Swollen lymph nodes (n = 5)	Painful genital lesions (n = 2)	Scabies (n = 2)	Fungal infection (n = 2)	*Any clinical sign * (n = 20)
Burning on urination (n = 33)	4	0	2	1	2	0	7
Frequent urination (n = 20)	4	0	1	0	0	0	4
Difficulty urinating (n = 7)	1	0	2	1	1	0	4
Strong urine (n = 4)	2	0	1	1	2	0	3
*Any urinary complaint *(n = 51)	8	0	3	1	2	0	11
Premature ejaculation (n = 6)	0	0	1	1	1	1	2
Urethral discharge (n = 6)	6	0	1	0	1	0	6
Genital itching (n = 5)	1	1	2	2	1	0	3
*Any reproductive health complaint *(n = 60)	10	1	4	2	2	1	15

There was a trend that men having extramarital sex and men having sex with prostitutes were more likely to have positive clinical findings than men not having extramarital sex (5/13 vs. 15/105; odds ratio 3.8, 95% confidence interval 1.1 to 13.0 and 3/6 vs. 17/112; OR 5.6, 95% CI 1.0 to 30.0, respectively). None of the eight men using condoms had positive clinical findings compared with 18% (n = 20) of the men not using condoms.

## Discussion

This study indicates that there is a high level of reproductive health problems among those men attending a reproductive health camp in a rural area of India. More than one in ten men had urethral discharge and over one third reported urinary problems. The high number of men reporting urinary symptoms is similar to that of an unpublished study in Uttar Pradesh, India [12]. However, a survey in Bangladesh reported pain passing urine in only 8% of men questioned [2]. In our study, 14% of unmarried men admitted to having sexual relations and 8% of married men reported extra-marital sex in our study, which lies within the expected range from other studies in Southern Asia [11,12].

The enthusiasm from men for involvement in reproductive health programmes has been reported elsewhere in Southern Asia [2,15]. Factors perceived to have maximised the attendance rate may have included the use of purely male staff at the camp, the promotion of a 'check-up' rather than just treatment, the consultation with local people and the placement of the camp in a local school (based on feedback from the health workers and peer educators). The attendees appear to be similar to the local population in terms of demographic factors such as marital status, education and income; however, the attendees may not be representative of all the men in the area. It is possible that men who thought they had a problem might be more likely to attend a reproductive health check-up; conversely, men who perceived that they had problems might be less likely to attend through fear. Studies maximising the generalisability of the sample would improve our understanding of the problems in this section of the population.

Wang [5] identified the level of responsibility that men hold for the consequences of their sexual behaviour as a priority area, and other researchers have put this emphasis primarily on condom use [3]. This study has shown that none of the men with clinical findings of reproductive health problems, nor any of the men visiting prostitutes, reported using condoms. Men who did not use a condom, who had sex with a prostitute or who had extramarital sex also appeared to be more likely to have clinical findings of reproductive health problems. The uptake of condom use was equally low compared to other reports, making the promotion of condom use in this population even more important [11].

The high proportion of men reporting urinary problems has also highlighted the need testing of urinary samples. One study in Bangladesh indicated that among 47 men reporting pain passing urine, only one was infected with Chlamydia trachomatis [2]; we are not aware of similar data for Indian samples.

A significant number of men did not complain of reproductive health problems (such as urethral discharge) during the interview, but had positive clinical findings (table 4). This incoherence has been reported in other studies [2,11], and could reflect a reticence about admitting to such problems, or that the men were unaware of their symptoms which may explain the low proportion of men seeking treatment for their problems [8]. Therefore, the dissemination of information about symptoms of reproductive health problems highlights another important area for further investigation.

There are some limitations to this study. First of all the numbers were relatively small and a larger sample size might have given further insight. Information obtained from urethral smears of all attendees may have provided important information. However, this was not possible in our setting due to technical problems. Further, not all areas of relevance to reproductive health were addressed in this study. Psychosexual problems have been commonly reported elsewhere [2], but were not asked about as part of this study. Some priority areas for male reproductive health, namely HIV/AIDS and male infertility [5], have not been addressed here. These areas were not addressed as CINI did not have sufficient resources, and there were no referral linkages to appropriate facilities at the time. Inclusion of these problems in future research is vital in improving overall reproductive health.

## Conclusions

This study provides important information about male reproductive health problems in a sample of men in rural West Bengal, India. The response of the men and the fact that nearly all attenders were willing to undergo a clinical examination is encouraging, and emphasises the possibilities for future research. The high level of reproductive health problems identified on clinical examination, but not reported in interviews, plus the low levels of condom use illustrate the need for reproductive health interventions with such groups. Future research could build on the findings of this exploratory study.

## List of abbreviations

CINI = Child in Need Institute

VDRL = Venereal Disease Research Laboratory

## Competing interests

The author(s) declare that they have no competing interests.

## Authors' contributions

KMD participated in the design of the study, performed the statistical analysis and drafted the manuscript. SD conceived the study, participated in its design, co-ordination, data entry and analysis and contributed to the manuscript. RD conceived the study, and participated in the design, co-ordination and analysis. All authors read and approved the final manuscript.
